# Exploring how patients understand and assess their diabetes control

**DOI:** 10.1186/s12902-018-0309-4

**Published:** 2018-11-06

**Authors:** Anjali Gopalan, Katherine Kellom, Kevin McDonough, Marilyn M. Schapira

**Affiliations:** 1 Division of Research, Kaiser Permanente Northern California, 2000 Broadway, Oakland, CA 94612 USA; 2 Michael J. Crescenz VA Medical Center, 3900 Woodland Ave, Philadelphia, PA 19104 USA; 3 Policy Lab, The Children’s Hospital of Philadelphia, 3401 Civic Center Blvd, Philadelphia, PA 19104 USA; 4 Division of General Internal Medicine, The Perelman School of Medicine at the University of Pennsylvania, 3400 Civic Center Blvd, Philadelphia, PA 19104 USA

**Keywords:** Diabetes, Qualitative research, Hemoglobin A1c, Glycemic targets, Doctor-patient relationships

## Abstract

**Background:**

Poor understanding of diabetes management targets is associated with worse disease outcomes. Patients may use different information than providers to assess their diabetes control. In this study, we identify the information patients use to gauge their current level of diabetes control and explore patient-perceived barriers to understanding the hemoglobin A1c value (HbA1c).

**Methods:**

Adults who self-reported a diagnosis of diabetes were recruited from outpatient, academically-affiliated, Internal Medicine clinics. Semi-structured interviews were conducted with participants and collected data were analyzed using thematic analysis.

**Results:**

The mean age of the 25 participants was 56.8 years. HbA1c was one of several types of information participants used to assess diabetes control. Other information included perceived self-efficacy and adherence to self-care, the type and amount of medications taken, the presence or absence of symptoms attributed to diabetes, and feedback from self-monitoring of blood glucose. Most participants reported familiarity with the HbA1c (22 of 25), though understanding of the value’s meaning varied significantly. Inadequate diabetes education and challenges with patient-provider communication were cited as common barriers to understanding the HbA1c.

**Conclusions:**

In addition to the HbA1c, several categories of information influenced participants’ assessments of their diabetes control. Increased provider awareness of the factors that influence patients’ perceptions of diabetes control can inform effective, patient-centered approaches for communicating vital diabetes-related information, facilitating behavior change towards improved patient outcomes.

**Electronic supplementary material:**

The online version of this article (10.1186/s12902-018-0309-4) contains supplementary material, which is available to authorized users.

## Background

Correct knowledge of diabetes management targets is associated with better glycemic control and improved diabetes self-care [1–4]. However, past studies estimate that as few as 25% of people with diabetes can accurately describe the meaning of the hemoglobin A1c value (HbA1c) or recall their most recent value [2, 5]. While this may not seem surprising given the conceptual complexity of the HbA1c value (e.g., expressed as a percentage, non-intuitive goal range), even simpler assessments of diabetes control appear difficult [6–8]. In two prior studies, individuals with poor glycemic control were asked to more generally describe their current diabetes control using Likert scales with qualitative descriptors. Many of these individuals, particularly those with low health literacy, erroneously described their diabetes as well-controlled in spite of average HbA1c values over 9% (11.7 mmol/L) [7, 8]. While these findings may simply indicate a lack of patient understanding of the HbA1c, they may also reflect differences in the ways patients and providers conceptualize and gauge diabetes control. Though providers may expect patients to also use the HbA1c value, the rubric used by patients to assess control may be different and remains incompletely understood.

In several established models of health behaviors and outcomes, individuals’ awareness and assessment of their current disease status are important predictors of behavior change or outcomes. Examples include perceived “disease severity,” cited in the Health Belief Model, the role of “consciousness raising” in the Transtheoretical Model of Health Behavior Change**,** where knowledge or information can contribute to a shift from the pre-contemplation stage to the contemplation stage, and the “informed, activated patient” in Wagner’s Chronic Care Model [9, 10]. Still, little remains known about the factors that contribute to these disease-related assessments for patients with diabetes. Better knowledge of the factors influencing patients’ evaluations of their diabetes may enable providers to communicate more effectively regarding diabetes management targets and current levels of diabetes control.

We conducted semi-structured interviews with patients with diabetes to address the following research questions: 1) *What information do patients use to assess their current level of diabetes control?* and 2) *What are patient-perceived barriers to understanding the HbA1c value?*

## Methods

### Study design and oversight

The study was approved by the University of Pennsylvania’s Institutional Review Board. Oral informed consent, including approval for audio-recording, was obtained prior to the start of each interview.

### Setting and participants

We recruited patients from the waiting rooms of two academically-affiliated Internal Medicine practices located in West Philadelphia. The individuals approached regarding participation were those that happened to have an appointment with a provider on a day we were recruiting (i.e., a convenience sample). Eligible individuals were at least 18 years of age and self-reported a diagnosis of diabetes. Pregnant women, non-English speakers, and individuals unable to verbalize understanding of the provided study information sheet were excluded. Participants received a $30 gift card to CVS (a common drugstore) for their participation.

### Data collection

Interviews, 30–60 min in length, were conducted by a research assistant (KM) and then transcribed verbatim. AG directly observed the initial interviews and regularly reviewed interview transcripts to provide feedback to KM on interview techniques. KM also took field notes. Sociodemographic information and diabetes history was collected from participants. The interview first focused on what information participants use to decide if their diabetes control is “good” or “bad” from day-to-day, month-to-month, and year-to-year. Participants were then asked if they were familiar with the HbA1c value. If they reported familiarity, they were asked what the value meant and about their most recent value. All participants, regardless of stated familiarity, were then read a short description: “*The hemoglobin A1c is a blood test that doctors use to measure how well a person is managing their diabetes. The test measures a person’s average blood sugar over the past 2-3 months. For most people with diabetes, the goal for the hemoglobin A1c is 7% or less.”* Participants were then asked how this description changed their previous understanding of their level of diabetes control. Next, participants were asked to perform an assessment of comprehension, referred to as the *Sharon* vs. *John HbA1c Comprehension Test*. They were presented with two written scenarios: 1) *“John is a 56-year-old man with a history of diabetes. His most recent hemoglobin A1c value was 9%”* and 2) “*Sharon is a 65-year-old woman with a history of diabetes. Her most recent hemoglobin A1c value was 6.8%.”* Participants were asked to select the individual (Sharon or John) that they believed had better diabetes control. The *Sharon* vs. *John HbA1c Comprehension Test* was done after, rather than before, the above description of HbA1c as it was intended to test participants’ comprehension and application of this provided information (i.e., not intended to test baseline HbA1c understanding). Finally, participants were told that many patients with diabetes have trouble understanding the HbA1c value and were asked for their thoughts on plausible reasons for this difficulty. The interview guide is included in the Additional file 1: Appendix.

### Analysis

**Fig. 1:**
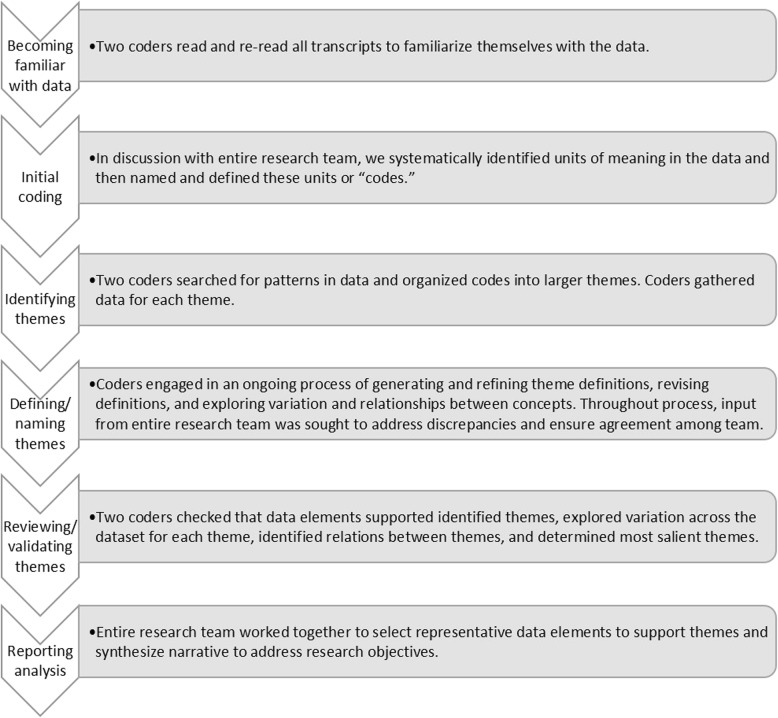
Overview of thematic analysis approach as described by Braun and Clarke [11]

Descriptive statistics were performed on the collected demographic and diabetes history data, as well as on participant accuracy on the *Sharon* vs. *John HbA1c Comprehension Test.* Participants’ interview responses (identified by study ID only) were analyzed using a thematic analysis approach as described by Braun and Clarke and summarized in Fig. 1 [11]. Two independent reviewers, KK (a qualitative methods consultant) and AG (a health services researcher with qualitative methods training), read and re-read the transcripts to familiarize themselves with the data. With input from the entire research team, units of meaning, or “codes,” within the data were then systematically defined. As additional transcripts were reviewed, these codes were organized into larger themes. The thematic definitions were refined and revised as the analysis proceeded, and variations and connections within the themes were noted and explored. The entire research team worked to determine the most salient themes pertaining to the research questions and to select the relevant data elements (participant [P] quotes) to synthesize a narrative addressing these questions. The reviewers independently coded transcripts until thematic saturation (no new themes emerging) was achieved (*N* = 20). The five remaining transcripts were then coded by AG only. KK and AG met to review coding comparison query results and discrepancies were resolved through study team discussion leading to consensus, with the coding scheme and definitions updated as needed. This process resulted in an average final inter-reviewer agreement of 97.6% (high inter-rater reliability was defined a priori as ≥90% agreement). NVivo Qualitative Analysis Software (QSR International Pty. Ltd., Version 10, 2014) was used to facilitate data management, coding, inter-rater reliability review, and analysis.

## Results

Between June 2013 and December 2013, approximately 240 individuals were approached regarding participation. Of those approached, 83 reported having diabetes, and 25 individuals agreed to participate. Of this 25, 68% were women and 84% were Black. There was a wide range of educational attainment, the mean duration of diabetes was 11 years, 36% of participants self-reported a diabetes-related health complication, and 40% reported current treatment with insulin (Table 1).

**Table 1:** Participant characteristics and diabetes history

Characteristic	*N* = 25	%
Age (mean years ± SD^a^)		57 ± 13
Gender		
Female	17	68
Ethnicity		
Hispanic	1	4
Race		
Black	21	84
White	1	4
Multiple^b^	3	12
Education		
Less than High School	3	12
High School or GED^c^	13	52
Some College/Technical School	6	24
College or beyond	3	12
Years since diabetes diagnosis (mean ± SD)		11 ± 7
Experienced a diabetes-related complication (Yes)	9	36
Diabetes Treatment		
Oral medications only	12	48
Insulin	6	24
Oral medications & Insulin	4	16
Diet only	3	12

^a^ *SD Standard deviation*

^b^Multiple = Individual 1: Black, White, and Asian, Individual 2: Black and Native American, Individual 3: Black and White

^c^GED General equivalency diploma

### Factors influencing participants’ assessments of diabetes control

Participants’ assessments of current control fell into the following thematic domains: 1) perceived self-efficacy and adherence to self-management; 2) the types and amount of medications taken; 3) the presence or absence of symptoms attributed to diabetes; 4) numerical data, both the HbA1c and self-monitoring of blood glucose (SMBG); and 5) connections between these domains.

1) Perceived self-efficacy and adherence to self-management

Of the 25 participants, seven referred to diabetes control in terms of self-efficacy, expressing confidence in their abilities to complete self-care and prioritize their health. For 17 participants, diabetes control was intrinsically linked with their perceived adherence to diabetes self-management behaviors. For the majority of participants (*n* = 17), self-care activities used in assessing diabetes control centered on maintaining a healthy diet, exercising, and weight management. In addition, four participants cited routine contact with providers as part of self-care used to assess their current diabetes control.

*I’m on top of my job…I’m on top of doing what I’m supposed to do to maintain this thing here. (P4)*

[Referring to poor control] *Not being disciplined, not setting my priorities and my priority is my body. (P9)*

*If I keep this excess weight off me. That’s how I’ll know* [about level of diabetes control]. *(P25)*

*I go to the doctor. Make sure that I get examinations for my eyes and for my feet, and come into my doctor regularly. (P18)*

2) The type and amount of medication taken

Perceived medication adherence, as well as the number and type of medications taken, affected ten of the participants’ perceptions of their current control. The ability to stop a medication (based on a physician’s recommendation) and not requiring insulin therapy were both considered markers of good control.

*When you’re not taking your medicine or doing what the doctors told you, you could tell by your health digressing. Like, more and more complications could arise or whatnot. (P13)*

*It’s well under control because I got a letter from my doctor telling me that my diabetes is well under control and if I wanted to stop the medication, I could. (P5)*

*I took insulin three times a day. And my doctor told me I’m doing good because I haven’t been back on insulin in ten years. (P8)*

3) Presence or absence of symptoms attributed to diabetes

Over half of the participants (*n* = 14) cited the presence of diabetes-related symptoms as an indicator of “bad” diabetes control. While eight participants mentioned specific symptoms often ascribed to hyperglycemia (e.g., polyuria, polydipsia, blurry vision), others mentioned symptoms not as readily attributable to diabetes, such as pain (*n* = 3) or general feeling of low energy and malaise (*n* = 9).

*Because, of course because you feel better, for one thing. And like I said, some of the things like I was doing, running to the bathroom frequently. (P15)*

*Because sometimes it be real high cause you get headaches and a lot of pain. Your bones ache and stuff and you’re in pain. (P21)*

*Things in your body that say, hey, it’s not right. (P18)*

*How do I tell if I’m in good control? When I don’t feel tired. (P7)*

4) Numerical data

Of the 25 participants, 16 referred to the HbA1c without prompting (i.e., prior to any mention of the HbA1c by the interviewer) as information used to assess diabetes control. However, seven patients did not refer to the value correctly by name (e.g., A1, AC1 U). Besides the current HbA1c, eight participants stated that improvements in the HbA1c over time were an indicator of good diabetes control. Nearly all participants (*n* = 21) also mentioned the use of SMBG as a means of assessing current diabetes control.

*Numbers—like when you take your sugar, hopefully all your numbers are where they’re supposed to be. (P1)*

*I have to do finger pricks. I take my—got machine where I take my diabetes, and that tells me what amount the diabetes is. (P16)*

5) Connections between domains

There were several commonly made connections between domains. Adherence to diabetes self-management or medications, together with numerical data (SMBG or HbA1c), was a combination that 11 participants used to assess their diabetes control. Another combination used by 13 participants to assess current control was numerical data (SMBG or HbA1c) along with the presence or absence of diabetes-attributed symptoms.

*I try to keep my weight down, but I’m picking up weight now. So I’ll be looking any day when I come to the doctor that she tells me that the count* [referring to the HbA1c] *is up again because I’m gaining weight. And I know that. (P8)*

*Doing well, yeah, by my finger sticks. And even when I go to the doctor, they check the blood through the arm. That’s when they told me my A1c was up. But now it’s down because I’ve been taking the medicine twice. (P7)*

*It’s mainly the hemoglobin A1c and just how I feel. (P22)*

### Exploring understanding of the HbA1c value

Although nearly all participants (*n* = 22) had at least heard of the HbA1c in the past, and the majority mentioned the HbA1c without prompting (*n* = 16), understanding of the value varied greatly. When asked what the value meant, six participants described what the test measured (e.g., an average over a period of time), ten participants described their general interpretation (both accurately and inaccurately) of the value (i.e., goal to be lower, target range), three participants described a more personal importance of the value, and three participants had just heard of the test before but could not provide further description. After being given the basic description of the HbA1c, seven participants reported that this information changed their previous understanding in some way.

*How much sugar was in my body for the last three months. The HbA1c measures for the last three months. And it’s supposed to be seven or below. But I keep mine at 6.2, once in a while at 6.4. Once it goes 6.4, I get back on my strict diet to knock it down to 6.2. I don’t like for it to pass 6.2. (P24)*

*No, I don’t know the meaning. All I know is it’s got to be low instead of high. (P21)*

*Like you could do your sugars every day and you could see it, but that’s the main number that shows that all of them numbers. (P3)*

*It means lot, it means that this is something that can affect my health if I don’t stay on top of it. (P10)*

*I know it is life saving for me and like it helps like it’s a super guideline. If it is off course, then my doctor can correct it through the medication. (P11)*

Several participants (*n* = 3) thought of the HbA1c as a sort of detector of non-adherence to diabetes self-management.

*And so, that level* [referring to the HbA1c] *let me know I was cheating, and I have to just get back on track. (P20)*

*Because that’s the only way that you can really find out if a person is really doing what they supposed to be doing as far as diabetes is concerned. (P4)*

#### John vs. Sharon HbA1c Comprehension Test

After hearing our description of the HbA1c, most participants (*n* = 22) accurately stated that Sharon (HbA1c = 6.8%) had better diabetes control than John (HbA1c = 9%). Most of the participants who answered Sharon stated that their choice was based on Sharon’s lower HbA1c value (*n* = 19), however, three participants explained their choice differently (based on Sharon’s age, based on decimal point) or doubted their choice (needed more information).

*Given she’s 65, I’m going with 6.8%. Because she’s 10 years older than John. And I don’t know, that was my analogy of it. (P15)*

*I want to say the 6.8, but you don’t know all of the factors that relate to why John’s is 9. He could be doing everything he’s supposed to be doing. He could have his weight under control and he could be one of those folks whose insulin levels is high. (P2)*

Of note, the three individuals who answered incorrectly were not the same three who reported no prior familiarity with the HbA1c. The reasoning provided by these individuals exposed general misunderstanding of the HbA1c and its interpretation.

*Because of the levels of it* [referring to the HbA1c]*. They’re supposed to be like I think seven, eight and nine. So I think his is in more control because I think nine is the top number or something, I think. (P5)*

#### Participant Perceived Barriers to Understanding the HbA1c Value

Participants’ thoughts on why the HbA1c might cause confusion for some patients fell into two main categories: 1) need for more diabetes-related education (*n* = 6) and 2) need for better provider communication of this information (*n* = 8).

*I think people need to be a little more education…just don’t give them the class and after they’re done, they go about their business. They really need a follow-up. (P2)*

*So I think education, if a diabetic is educated from the door* [referring to the time of diabetes diagnosis]*, it would be more beneficial. (P20)*

*I think people psych their selves out listening to medicalese. Like to me, the best doctors are doctors that can make it plain. (P12)*

*They’re* [referring to patients] *scared of the doctors. They think they* [referring to doctors] *went to school all this time, they know what they’re talking about. (P8)*

## Discussion

While the HbA1c was an important contributor to participants’ evaluations of their diabetes control, participants also considered other information, including perceived self-efficacy and adherence to self-care activities, the types and amounts of medications used, the presence or absence of symptoms attributed to diabetes, and SMBG.

The importance participants placed on diabetes self-efficacy and self-care activities is well-founded and supported by existing literature [12, 13]. Provider awareness of the roles that self-efficacy and self-care activities play in patients’ model of diabetes control is vital. By focusing on building self-efficacy, and supporting and reinforcing self-care in their communications with patients, providers’ recommendations and feedback can be more in line with patients’ perspectives and priorities, hopefully increasing the effectiveness of these communications [14].

Medications, particularly the use of insulin, were linked to many participants’ perceived diabetes control. The belief that more medications or insulin use is an indicator of worse or “end-stage” disease is established in the current literature and is a common barrier to medication intensification, particularly insulin initiation [15, 16]. Dispelling the idea that insulin use is an indicator of current diabetes control and addressing beliefs regarding the number of medications taken could shift patients’ perceptions regarding diabetes control. Re-framing the types of pharmacologic treatments prescribed not as failures of management, but rather as results of individual physiologic differences, could have a beneficial impact on patient-provider communication, patient self-efficacy, and adherence to these prescribed medications.

Associations of patient-reported symptoms and symptom severity with diabetes outcomes, including patients’ self-rated health status and measured glycemic control, have been noted in past work [17, 18]. Although symptoms can be an indicator of poor glycemic control, two potential issues complicate patients’ use of this metric to gauge their current diabetes control. First, patients and providers may differentially attribute certain symptoms to diabetes. Patients may attribute symptoms such as general malaise and fatigue to poorly controlled diabetes, even though these are symptoms providers may not immediately associate with this disease [19, 20]. Notably, malaise and fatigue may actually be symptoms of depression, a condition that disproportionately affects patients with diabetes, remains underdiagnosed in this population, and may contribute to worse diabetes outcomes [21]. Second, the absence of symptoms may provide false reassurance to patients regarding their current diabetes control. Many patients with blood glucose levels well above goal do not experience any symptoms in spite of their increased risk for future diabetes-related complications. Providers should emphasize this point to patients in their communications about diabetes control.

Numerical information, either SMBG or the HbA1c, was part of many participants’ assessment of diabetes control. The frequent mention of SMBG as an important source of information is noteworthy given mixed evidence regarding the reported clinical value of ongoing SMBG in patients not on insulin (only 40% of interviewed participants reported insulin use) [22]. The importance given to the HbA1c in assessing control and management was somewhat surprising given the varying levels of actual understanding of the value’s meaning. Given past evidence supporting the value of an accurate understanding of disease management targets, like the HbA1c, further efforts are needed to improve the way care providers present this information to patients with diabetes [2–4]. Improving providers’ communication of this value could address the current barriers to HbA1c understanding and, hopefully, increase the HbA1c’s informational value for patients with diabetes. Further work is needed to identify optimal approaches for communicating information on glycemic control to patients. Towards this goal, in an in-progress mixed-methods study, we elicited input from participants with diabetes on a variety of visual formats for presenting the HbA1c value (e.g., color-based scales, depictions of distance from goal). Based on qualitative analysis of participants’ input, two formats were chosen for testing against standard presentation of the HbA1c in a three-arm randomized, controlled trial of patients with diabetes to assess their impact on patients’ assessments of their current diabetes control.

The study has several key limitations. First, the population is small and demographically homogenous, limiting the generalizability of the findings to other populations. Of note, the race/ethnic demographics of the participants do reflect that of West Philadelphia, a predominantly Black neighborhood. Because demographic information was not collected from eligible individuals who declined participation, differential participation by race or educational attainment cannot be assessed. Second, while the investigator AG’s supervision and coding of the initial interviews may have introduced potential bias, it was necessary for the interviewer’s (KM) training and unavoidable given limited study staff. We feel that the use of a structured interview script, coding by two independent individuals, and input from the broader study team during the analysis process, helped to mitigate this potential bias. Third, participants willing to take part in this type of interview may be more engaged and activated than the typical patient with diabetes. Finally, we did not ask participants to specify whether they had Type 1 or Type 2 diabetes. In our clinical experience, many patients are not sure of their diabetes type and, in the absence of blood tests (i.e., insulin autoantibodies, c-peptide), providers cannot always be entirely certain of diabetes type among patients treated only with insulin. However, the age of participants and use of oral medications or diet for management suggest most participants would be classified as having type 2 diabetes.

## Conclusions

In this qualitative exploration of how patients with diabetes gauge their level of diabetes control we identified several types of information used by participants to assess their diabetes control. Most participants correctly emphasized the role of medication adherence and a healthy lifestyle in diabetes management and accurately equated lower HbA1c and blood glucose values with good diabetes control. However, many participants inaccurately believed that the type and amount of medication taken was an indicator of diabetes control and felt falsely reassured by the absence of diabetes-related symptoms. Provider awareness of the factors influencing patients’ assessments of their diabetes control may help providers communicate more effectively with patients about their diabetes management status and targets, towards the goal of improved outcomes.

## Additional file

Additional file 1: **Appendix** Interview Guide for Semi-Structured Interviews. (DOCX 16 kb)

## Abbreviations

Hemoglobin A1c: HbA1c
P: Participant
SD: Standard deviation
SMBG: Self-monitoring of blood glucoses
