# Supplementary material for: Exploring how patients understand and assess their diabetes control
Source: BMC Endocr Disord. 2018 Nov 6;18:79. doi: 10.1186/s12902-018-0309-4 (PMC6219190; doi:10.1186/s12902-018-0309-4)
Supplement: Supplementary file 1 — Appendix Interview Guide for Semi-Structured Interviews. (DOCX 16 kb) [file 12902_2018_309_MOESM1_ESM.docx]

**Interview Guide for Semi-Structured Interviews**

**Introduction**

The goal of this interview is to discuss ways that doctors can improve the way we talk to our patients about their level of diabetes control. We have found that many patients have trouble understanding whether or not their diabetes is well or poorly controlled.

We are trying to come up with new ways to present information about current diabetes control to our patients. We think that if we make it easier for people to understand how they are doing with their diabetes management, it will be easier for them to make better decisions about their health.

**Diabetes Control**

How well controlled do you think your diabetes is right now?

What information do you use to decide if you are meeting your goals for managing your diabetes? From year-to-year? Month-to-month? Week-to-week? Day-today?

How important do you think it is to control your diabetes? Tell me why you think that.

How does your doctor communicate with you about your diabetes care in general?

About letting you know if you are reaching your goals?

About letting you know if you are in good control?

In what ways does your doctor do a good job in communicating these points?

In what ways could your doctor’s communication be improved?

Are you familiar with the hemoglobin A1c value?

What does this value mean to you?

What is your most recent result?

What do you think this result says about your current diabetes control?

**Reviewing the Hemoglobin A1c**

- **For those who stated they were familiar with the A1c:**

You stated you are familiar with the hemoglobin A1c, but just as a review, the hemoglobin A1C is a blood test that doctors use to measure how well a person is managing their diabetes. The test measures a person’s average blood sugar over the past 2-3 months. For most people with diabetes, the goal for the hemoglobin A1C value is 7% or less. Higher values over a period of time are associated with more complications from diabetes such as kidney disease, heart disease, and strokes

After hearing this description of the hemoglobin A1c, what questions do you have about this value and its meaning?

How did what I just tell you about the hemoglobin A1c affect your understanding of how you are doing managing your diabetes?

Many patients have trouble understanding the hemoglobin A1c value and how it relates to their diabetes control. Why do you think this value may be confusing to some people?

- **For those who stated they were not familiar with the A1c:**

The hemoglobin A1c is a blood test that doctors use to measure how well a person is managing their diabetes. The test measures a person’s average blood sugar over the past 2-3 months. For most people with diabetes, the goal for the hemoglobin A1c is 7% or less.

After hearing this description of the hemoglobin A1c, what questions do you have about this value and its meaning?

How did what I just tell you about the hemoglobin A1c affect your understanding of how you are doing managing your diabetes?

Many patients have trouble understanding the hemoglobin A1c value and how it relates to their diabetes control. Why do you think this value may be confusing to some people?

- **For both of the above groups**

Based on the information I just told you about the hemoglobin A1c, I would like to describe two pretend patients and get your opinions on the level of their current diabetes control.

*John is a 56-year-old man with a history of diabetes. His most recent hemoglobin A1c value was 9%.*

*Sharon is a 65-year-old woman with a history of diabetes. Her most recent hemoglobin A1c value was 6.8%.*

Of the two people I just described, which one’s diabetes would you say is better controlled? Why?
